# Uncovering Barriers and Facilitators of Weight Loss and Weight Loss Maintenance: Insights from Qualitative Research

**DOI:** 10.3390/nu15051297

**Published:** 2023-03-06

**Authors:** Audrey Tay, Hannah Hoeksema, Rinki Murphy

**Affiliations:** 1 Faculty of Medical and Health Sciences, University of Auckland, 22-30 Park Avenue, Auckland 1142, New Zealand; 2 Te Toka Tumai Diabetes, Te Whatu Ora Health New Zealand, 214 Greenlane West, Auckland 1051, New Zealand

**Keywords:** weight loss, weight loss maintenance, barriers, facilitators, qualitative, perspectives

## Abstract

Long-term weight loss maintenance is often difficult to achieve. This review analysed qualitative data on self-perceived barriers and facilitators of weight loss and weight loss maintenance among weight loss intervention participants. A literature search was conducted using electronic databases. Qualitative studies written in English and published between 2011–2021 were eligible for inclusion if they explored the perspectives and experiences of individuals who received standardised dietary and behavioural support for weight loss. Studies were excluded if weight loss was achieved through self-directed methods, only increasing physical activity, or surgical or pharmacological interventions. Fourteen studies were included, totaling 501 participants from six countries. Thematic analysis was used to identify four aggregate themes: internal factors (i.e., motivation and self-efficacy), programme-specific factors (i.e., the intervention diet), social factors (i.e., supporters and saboteurs), and environmental factors (i.e., an obesogenic environment). Our findings demonstrate that internal, social, and environmental factors all influence weight loss success, as well as the acceptability of the weight loss intervention. Future interventions may be more successful if they prioritise participant acceptability and engagement by, for example, providing tailored interventions, a structured relapse management plan, strategies to enhance autonomous motivation and emotional self-regulation, and extended contact during weight loss maintenance.

## 1. Introduction

Rates of obesity have increased worldwide [1,2]. Obesity is associated with increased risk of chronic diseases, including type 2 diabetes [3], fatty liver disease [4], polycystic ovarian syndrome [5] and cardiovascular disease [6]. Losing excess body weight can help prevent and manage these conditions [7,8,9,10], but weight loss can be difficult to sustain over the long-term due to both behavioural and biological compensatory factors that promote weight regain [11,12,13,14], such as changes in appetite and energy expenditure [15,16,17]. Bariatric surgery is an effective treatment for rapid initial and sustained weight loss [16,18], but it may not be suitable for all individuals [19] and may not guarantee long-term weight maintenance in the context of poor dietary habits and physical inactivity [20,21].

Some individuals can lose weight through lifestyle interventions and maintain this over the longer term. Previous research has identified factors associated with weight loss success, such as achieving a greater initial weight loss [22,23], initiating weight loss after a medical event (such as a heart attack) [24,25,26], adhering to diet and exercise strategies [22,24], regular self-monitoring [27,28,29,30], having better mental wellbeing [22], and preventing small weight gains from becoming more significant [31,32]. These factors suggest that psychological and behavioural factors play a significant role in weight loss and weight loss maintenance over time.

Understanding individuals’ experiences when participating in weight loss interventions can provide insights into how to design more effective and sustainable interventions [33]. However, there is limited qualitative evidence on the barriers and facilitators of weight loss and weight loss maintenance from the perspective of intervention participants. Most of the current literature has focused on participants’ experiences after the study ends, and these findings may not be generalisable as they are limited to a single programme in a specific context. One systematic review [34] identified key psychological, socio-cultural and environmental mediators of weight loss. However, it included studies published between 1990 and 2010, and there was no consistent participation in weight management interventions among study participants.

This review aims to expand on previous research and identify key themes related to the barriers and facilitators of weight loss and weight loss maintenance by analysing qualitative data published between 2011 and 2021. Studies were eligible for inclusion if they reported data from participants in a weight loss intervention that used dietary manipulation to achieve weight loss. The review will focus on participants’ experiences and perspectives during the weight loss or weight loss maintenance phase. By including more recent studies, this review aims to gain a more contemporary understanding of participants’ perspectives in order to inform the development of more effective weight management interventions in future.

## 2. Materials and Methods

The aim of this review was to synthesise qualitative evidence on the barriers and facilitators of weight loss and weight loss maintenance in overweight or obese adults who received standardised dietary and behavioural support. We conducted a literature search from 1 March 2021 to 31 July 2021 using a range of electronic databases, including PubMed, Medline, EMBASE, Web of Science, and Scopus. The search used the following words: ‘barriers’, ‘facilitators’, ‘weight loss’, ‘weight loss maintenance’, and ‘qualitative’. Qualitative studies were eligible if they were written in English, published between 2011–2021, and reported on the attitudes, perspectives, and experiences of participants who received standardised dietary and behavioural support for weight loss or weight loss maintenance. Studies were excluded if they used self-directed methods, only increased physical activity, involved bariatric surgery or pharmacological interventions, or only investigated the perspectives of healthcare professionals or family members.

Two reviewers (AT and HH) independently screened the titles and abstracts of the studies identified by the search strategy, and then screened the full-text articles for eligibility. A modified version of the Critical Appraisal Skills Programme (CASP) quality assessment tool for qualitative studies [35] was used to evaluate the quality of the included studies. Data on participant and intervention characteristics and data collection and analysis methods were extracted from each article and summarised in a table.

The full-text articles were imported into NVivo11 (QSR International, 2015) for thematic analysis. The reviewers read each article several times and used an open coding framework to assign codes to individual words or phrases that represented relevant concepts. The coded data was organised into first-order themes, which were reviewed and confirmed by the two reviewers. Using thematic analysis, the reviewers identified aggregate dimensions by analysing the data across studies to highlight common themes. The two reviewers agreed on the aggregate dimensions that best reflected the literature content. An inductive approach was used to interpret the data and draw conclusions based on the identified first-order themes and aggregate dimensions.

## 3. Results

**Figure 1:**
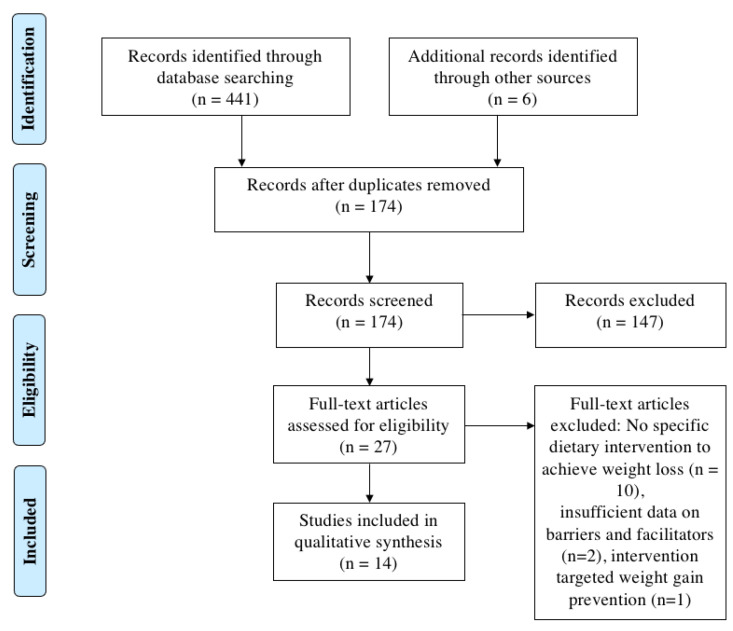
PRISMA diagram outlining the process of selecting studies for inclusion in this review.

The literature search process is outlined in Figure 1. A total of 441 references were identified through the literature search, which was reduced to 174 articles after eliminating duplicates. Of these, 152 studies did not meet the inclusion criteria as they included patients who had undergone bariatric surgery, included children or adolescents, focused on the perspectives of healthcare providers or carers, only used an exercise intervention to achieve weight loss, or were not a qualitative study. The reference lists of potentially relevant papers were also searched. A total of 27 full-text articles were assessed for eligibility, and ten of these were excluded because the participants underwent self-directed weight loss. Two additional studies were excluded because there was insufficient material on barriers and facilitators of weight loss or weight loss maintenance. One study was excluded as the intervention focused on weight gain prevention instead of weight loss.

Fourteen qualitative studies were included in this review. Six of these studies focused on participants’ experiences during the weight loss phase, and eight examined the experiences of both weight loss and weight loss maintenance. Five of the studies were nested within randomised controlled trials. One study included the views of both study participants and dietitians; however, only the participants’ perspectives were included in this analysis. A summary of the included studies is presented in Table 1.

The quality of included studies was mixed, as shown in Table 2. Items on the CASP were appraised as “unclear” if the information was absent or insufficient to judge its quality. All of the studies collected data in a way that addressed the research question, but two studies did not disclose or consider potential researcher bias.

Four aggregate dimensions emerged from the data: internal factors, programme factors, social factors, and environmental factors. Internal factors refer to barriers and facilitators related to personal attributes, motivations, and attitudes towards weight loss. Programme factors include the design, delivery, and features of the weight loss intervention itself, and how they impact adherence and outcomes. Social factors encompass interpersonal influences outside of the weight loss intervention, such as social support and family dynamics. Environmental factors focus on external factors that may influence weight loss outcomes. Evidence to support these aggregate dimensions are presented in Table 3.

### 3.1. Internal Factors

#### 3.1.1. Motivation

Motivation was a key theme in the studies reviewed. Although baseline motivation was not formally assessed, it was frequently mentioned as a facilitator of weight loss success. Participants often entered the study motivated by a desire to improve their health, either because they had a pre-existing health condition [38,40,41,49], were at risk of developing chronic health conditions [36,47], or wanted to prevent the recurrence of disease [48]. Participants also reported being motivated by personal values, such as wanting to live longer, participate in important relationships, and set a positive example for loved ones [40,41]. Enrolling in an intervention study was also seen as a sign of motivation and readiness to make lifestyle changes [38,46,47,48].

During the interventions, the source of motivation appeared to shift. Weight loss success further motivated participants to continue with the intervention [38,39,42,45,49], and this theme was particularly evident in studies that used meal replacements due to the rapid weight loss [41,45,47]. However, participants who had unmet weight loss expectations quickly lost motivation and were less likely to sustain behaviour changes [39,41]. Other drivers of motivation included improvements in clinical parameters [38,40,49], self-reported quality of life [44,47], and physical attractiveness [41]. Regular physical activity also increased motivation and facilitated dietary changes [42,44,45,49]. For some participants, extrinsic drivers of motivation, such as following a structured plan [36,39,42,44,45] and being involved in a research study [40,41,47,49], were important in driving behaviour change. However, these extrinsic motivators were not enough to sustain changes once the study ended, and adherence and motivation often declined. Therefore, motivation was perceived as a facilitator of weight loss, but not weight loss maintenance.

#### 3.1.2. Self-Efficacy

Self-efficacy, or the belief in one’s ability to successfully implement new dietary habits, was crucial for adopting healthy behaviours and achieving weight loss [36,39,40,41,46,47]. Many participants were initially motivated to lose weight, but reported low self-confidence [46,49] due to past unsuccessful weight loss attempts [40,41]. Additionally, participants often struggled with emotional regulation and used food as a source of comfort, hindering their ability to adopt healthy behaviours [36,40,41,43,46,48,49]. One participant stated, “When something happens in my life, things I cannot influence or that I find difficult, it’s very easy for me to find my way to the fridge” [38]. These perceptions of low self-efficacy were mainly reported retrospectively and were not formally assessed in the included studies.

Several factors influenced perceived self-efficacy. Some participants found the diet easier to follow than expected, which increased their adherence self-efficacy [40,46]. One participant said, “Participating in the study has changed me. I thought before that I was the kind who couldn’t get slim, but today I realise that it is quite easy to influence, with the right diet” [46]. In addition, previous performance contributed to self-efficacy. For example, those who successfully handled past challenges, such as attending an event where food was present and not deviating from the diet, were more equipped to overcome other difficult situations [40]. Setting clear goals and regular self-monitoring also reinforced perceived self-efficacy and facilitated behaviour change [38]. Participants with higher self-efficacy were more likely to take self-motivated steps towards weight loss and weight loss maintenance, such as restructuring their food environment, adopting healthier habits, and seeking additional external support when the study ended [40,41].

### 3.2. Programme Factors

#### 3.2.1. The Intervention Diet

The convenience of the diet or weight loss programme was a key factor that influenced adherence, particularly in studies which used meal replacements as they required little forethought and food preparation time [37,41,44,45,47]. One participant said, “Well a simpleton could do it…Add cold water to this and that’s it” [41]). However, some participants found the meal replacements unpleasant, tedious or monotonous due to the limited variety of sachet flavours and the absence of solid food. Some were able to overcome these issues by keeping in mind that the intensive intervention was relatively short-term (e.g., eight weeks) compared to the potential long-term benefits. Others, however, cited these issues as a strong reason for discontinuing the diet [41].

During the weight loss maintenance phase, participants struggled with the transition away from meal replacements. Quotes from participants include: “When you are in the [weight loss] phase it’s four shakes, your protein, vegetables, and your bar and you don’t have to think about it. Then you switch to the [weight maintenance] phase, and there’s a lot of decision making throughout the day”, “We have to figure out how to live without meal replacements. Now that we’ve lost the weight and kept it off, how are we going to adjust back to life without meal replacements?” [44]. The weight loss maintenance phase required more time and effort, and some participants found it difficult to adopt long-lasting routines during this crucial adaptation process [44].

Five studies used prescriptive caloric and exercise targets (without meal replacements) [40,42,46,48,49] and two recommended a particular dietary pattern (i.e., Paleolithic and Mediterranean diet) [38,39] with no calorie restriction. Regardless, participants reported similar challenges, such as struggling to follow the recommended foods, especially if their pre-intervention diet significantly differed from the intervention diet [39,40]. Inadequate variety [39,49], cost [36,39,40,49], and a yearning for ‘forbidden’ foods [39,49] were also barriers to adherence. On the other hand, two studies focused on healthy eating education, self-monitoring and setting regular goals, with no caloric or exercise targets [36,43]. These studies reported less programme-specific barriers.

#### 3.2.2. Supervision and Accountability

Continued supervision or accountability through regular weigh-ins, group meetings, and phone calls played a significant role in the success of weight loss interventions [36,42,44,45,46,47,48,49]. Personalised support [38,41,45,48,49] and accountability [36,41,42,44,45,46,49] were highly valued by participants and helped establish trust in a healthcare professional, which was a key factor in aiding success [36,37,38,39,43,44,45,48]. The type of personnel providing behavioural change support varied, including primary care nurses [36], a multidisciplinary team [37,38,41,42,43,45,47], health coaches [44], and dietitians [39,46,48,49]. Ultimately, the therapeutic relationship seemed to have a greater impact on the success of an intervention, rather than the therapy itself.

Participating in a research study involved external accountability and supervision, which was viewed as a key facilitator of weight loss. However, the discontinuation of supervision when the study concluded became a significant barrier to weight loss maintenance. After the intervention, participants reported feeling “set adrift” or in a “free fall” [47]. One participant stated, “You go from intensive supervision to no supervision at all at the conclusion of the programme. You don’t have regular weigh-ins or anything like that afterwards. The weigh-ins and that sort of thing are incentives during the study. Left to your own devices, you don’t have that to look forward to and tend to let things slide” [49]. Another participant emphasised the importance of ongoing contact, saying, “While I was actively on the program I did very well and lost weight. And to my distress it’s come back. I really think it was the regular contact with someone, because I didn’t want to a) let myself down and b) let the program down and my mentor down” [48]. For some participants, ongoing support and accountability was essential for long-term success [46]. Two studies provided extended virtual contact beyond the initial study intervention and reported sustained weight loss among those with continued engagement [37,48]. Some participants from other studies sought out additional support on their own, recognising their need for ongoing accountability [42]. However, for others, limited external accountability after the study became a barrier to maintaining weight loss long-term [49].

### 3.3. Social Factors

#### 3.3.1. Support from Others

Support from friends, family members, or work colleagues was mentioned in most of the studies as a key facilitator for weight loss success [36,39,40,41,43,45,47,48]. In particular, support from a partner or spouse was highly valued [39,42,43]. Friends and family members who considered the participants’ needs during social events [39,40] and complimented their appearance also provided valuable support during the weight loss journey [41]. The importance of peer support was evident in the Counterbalance study, where half of the participants were ‘buddies’ to each other or had a ‘diet buddy’ who was not involved in the study [41]. Furthermore, incorporating a group element in the intervention appeared advantageous as participants drew strength and inspiration from others [36] and gained a sense of community [47]. For some, the group also provided a competitive arena [47]. Although participants had varied connections with the group [44], they still valued the opportunity to participate in ongoing discussions about the weight loss process. This highlights the importance of normative social support and shared goals in diet adherence and weight loss success.

#### 3.3.2. Saboteurs

The influence of friends and family on weight loss efforts could be both positive and negative. Although some helped maintain healthy eating habits, some acted as saboteurs by pressuring participants to eat unhealthy foods [39,46,47] or making negative comments about their food choices, such as “You look ill”, “You don’t need to lose weight”, “You are having a salad again today?”, “I don’t know why you have to eat all that [healthy] stuff, just eat less”, and “You should stop losing weight” [46].

Social expectations and cultural norms also made it difficult for participants to adhere to the diet. A New Zealand study highlighted cultural expectations around food [36]. Food was described as ‘a blessing and not a blessing’, particularly for Māori and Pacific people, as food is central to meaningful social engagement and a source of cultural pride. Thus, refusing offered food is considered offensive. Some participants established explicit strategies to deal with external influences, such as bringing their own food to social events [36]; however, these social drawbacks strongly challenged weight loss efforts for many participants.

### 3.4. Environmental Factors

Geographical location and access to resources played a role in weight loss success. Some participants had good transport links and easy access to healthy foods [38], whereas others did not [36]. Participants noted difficulties managing an obesogenic environment [38,41,42], such as one participant who commented on the struggle to avoid tempting smells and sights, saying “I was in town at one point, bakeries everywhere and, it was ridiculous, I couldn’t concentrate…I would have been fine if I had been at home, I would have lost weight this week, and I would have still been on it, but I couldn’t stick to it” [41].

Additionally, certain environmental factors appeared to more commonly hinder weight loss maintenance compared to initial weight loss. For example, two studies provided access to exercise facilities during the weight loss intervention. However, after the study, some participants had limited access to exercise facilities which prevented regular physical activity [37,49]. One participant commented on the difficulty in finding a similar programme to the one used in the study with monitoring, stating, “They [commercial gyms] tend to leave you to your own devices or push a programme of their own” [49].

Five studies in this review focused on participants’ experiences during the weight loss phase [36,37,38,40,41]. It is unclear whether participants developed the necessary skills and habits to maintain weight loss in their environment. However, those who were less committed to behaviour change strategies [38,42,47], prioritised other things [39,42,49], or believed their obstacles were ‘insurmountable’ [43] were less likely to be successful in the face of environmental barriers. These findings suggest that successful weight loss and maintenance depended on managing external influences rather than being controlled by them.

## 4. Discussion

Weight loss maintenance is complex and requires a wealth of self-regulatory resources amidst a continual battle against biological and behavioural drivers of weight regain [50,51,52]. This review analysed the experiences of over 500 participants across 14 studies from various countries, uncovering key themes related to barriers and facilitators to weight loss and weight loss maintenance. Consistent with prior research [34], our findings demonstrate that individual, social, and environmental factors all influence weight loss success. However, this review also revealed that the acceptability of the intervention can play a critical role in determining weight loss success. These findings, therefore, have practical implications for the development of effective weight management programmes, highlighting the importance of participant acceptability and engagement.

Our findings suggest that motivation can be influenced by a range of factors, including personal values, health concerns and extrinsic motivators. Although initial motivation can be important in driving behaviour change, it may not be sufficient to sustain long-term changes [53]. In the reviewed studies, motivation and acceptability of the diet waned over time, which led to poorer dietary adherence. This phenomenon is common in weight loss interventions, even if participants can freely choose their weight loss intervention [54,55]. To address declining motivation, tailoring interventions to individual needs may be necessary [56,57]. This could be achieved by exploring barriers and facilitators pre-intervention to inform the type, quantity and intensity of support required at an individual level. For example, in this review, participants lacked confidence in their ability to succeed due to previous failed attempts [40,41,42]. Therefore, breaking the intervention into smaller, more manageable changes may have boosted adherence and success [58,59,60]. On the other hand, previous lifestyle interventions have used a two-week behavioural run-in period to identify less-motivated individuals before randomisation [61,62]. In this case, using a personalised approach could help less-motivated individuals recognise key barriers and formulate potential solutions.

Additionally, using behaviour change strategies that are driven by internal factors rather than external rewards or incentives may be more effective in achieving weight loss success [63]. Motivational interviewing, which focuses on helping individuals develop more autonomous motivation driven by personal values, interests, and enjoyment is one example of a technique that has been shown to be effective in increasing motivation and promoting weight loss [64,65,66,67]. Only one study in this review reported using motivational interviewing [45]; however, the study was not designed to measure the technique’s true effect. Regardless, although rapid weight loss was highly rewarding and increased motivation, encouraging individuals to cultivate more autonomous motivation may have facilitated long-term weight loss success [45,68].

The ability to regulate emotions was a commonly reported barrier [36,40,41,42,43,46,48,49] and may have been a strong contributing factor to weight regain. Managing difficult or unwanted thoughts and feelings is crucial for long-term weight loss success [69,70], yet many weight loss interventions do not address this aspect of behaviour. As recommended by previous research [34], future weight management interventions should help individuals recognise and manage psychological obstacles such as maladaptive behaviours and emotional regulation. Mindfulness and acceptance-based approaches, such as acceptance commitment therapy (ACT), provide a potential avenue for weight loss interventions [71,72]. In this context, ACT aims to promote healthy behavioural patterns consistent with one’s values, which involves teaching mindfulness strategies and self-acceptance, thereby enhancing the ability to take values-based action in the presence of unwanted thoughts, feelings and bodily sensations [71]. Pilot studies have shown some promising results in yielding superior weight loss outcomes [73,74,75], but more research is needed to support its effectiveness.

Successful weight loss may require different behaviours than successful weight loss maintenance [76]. By recognizing this, weight loss interventions can be designed with this premise in mind, potentially leading to more effective strategies for maintaining weight loss over time. The lack of external accountability and support after the initial weight loss intervention was considered a significant barrier to weight loss maintenance [39,44,46,48]. In contrast, providing extended contact may have effectively reinforced behaviour changes made during the initial weight loss intervention. One study in this review provided virtual contact for six months after the intervention through tailored text messages and reported sustained weight loss [48]. This concept is supported by evidence from a small number of studies showing that delivering extended contact post-intervention via text message can yield significant weight reductions compared to no extended contact [77,78]. Further, a systematic review of thirteen randomised controlled trials found that ‘extended care’ interventions, such as in-person group meetings or telephone calls, resulted in an additional maintenance of 3.2 kg over 17.6 months compared to interventions with no or minimal additional contact [79]. Thus, interventions should provide extended contact to reinforce behaviour changes made during the initial weight loss intervention using low-cost and widely available approaches, such as text messaging or group meetings. In fact, previous research suggests ongoing support for at least 2 years post-intervention [23], further highlighting its place in promoting weight loss maintenance. A formalised ‘rescue plan’ can also be helpful for managing weight regain, as shown in the DiRECT trial where almost half of the participants required this additional support [7].

Environmental factors appeared to be more commonly seen as a barrier to weight loss maintenance compared to the initial weight loss [36,38,41,49]. For example, insufficient exercise post-intervention inhibited weight loss maintenance as participants no longer had the same access to exercise opportunities [37,49]. Regular physical activity is crucial to prevent weight regain [80,81] and demonstrates the importance of supportive environments to encourage long-term healthy behaviours. To address this, interventions should ensure continued exercise support during weight loss maintenance, such as providing access to exercise facilities or equipment, and forming partnerships with local exercise organisations.

There are several methodological issues that may limit the findings of this review. Qualitative data was sourced from self-selected or purposive samples, which ranged from 4% to 95% of the primary intervention’s study population. Half of the studies (*n* = 7) were shorter than six months, and only three included those who dropped out of the intervention. Therefore, important perspectives among unsuccessful or frustrated individuals may have been missed. This review also focused on retrospective accounts, with only one study collecting qualitative data both pre- and post-intervention [41]. The results may therefore reflect post hoc rationalisation of events and be subject to recall bias.

Although the present study aimed to investigate both the barriers and facilitators of weight loss and weight loss maintenance, it is important to note that more data was collected on barriers and facilitators of achieving initial weight loss, compared to weight loss maintenance. This may have limited the extent to which the study was able to capture the full range of factors influencing weight loss maintenance. However, the study provides valuable insights which may still be useful in informing interventions designed to promote weight loss maintenance.

Overall, our findings build on previous research that have identified barriers and facilitators of weight loss from the perspective of overweight and obese adults. Dietary manipulation can achieve a calorie deficit, but several factors influence weight loss and weight loss maintenance success. Future interventions may be more successful if they provide tailored interventions based on individual needs, a structured relapse management plan, strategies to enhance autonomous motivation and emotional self-regulation, and extended contact during weight loss maintenance.

## Figures and Tables

**Table 1:** Study characteristics.

Author, Date	Country	Participants	Dietary Intervention	Behavioural Support	Weight Loss in Primary Intervention	Data Collection	Data Analysis
Studies focusing on weight loss							
Abel et al., 2018 [36]	New Zealand	Adults with newly diagnosed prediabetes (*n* = 20)	6 months of education on healthy eating principles with no specific calorie reduction advice.	3 times over 6 months plus 6 weekly group education sessions.	At 6 months: Intervention −1.3 kg, Control +0.8 kg (difference *p* < 0.001).	Semi-structured interviews	Thematic analysis
Fazzino et al., 2016 [37]	United States of America	Rural breast cancer survivors (*n* = 186)	6 months of 2 meal replacement shakes and at least 5 fruits of vegetables per day, plus 225 min of physical activity per week	Six months of weekly one-hour group teleconference calls	At 6 months: −12.8± 6.8% (*p*-value NA)	Semi-structured interviews	Thematic analysis
Haigh et al. 2019 [38]	England	Adults with non-alcoholic fatty liver disease (*n* = 19)	12 weeks of the Mediterranean diet, with no specific calorie reduction advice.	Single session of nutrition counselling and education at baseline.	At 12 weeks: 99.2 ± 17.0 kg at baseline to 96.8 ± 17.5 kg (*p* = 0.001)	Semi-structured interviews	Thematic analysis
Hammarström et al., 2014 [39]	Sweden	Post-menopausal females (*n* = 12)	2-year RCT of Paleolithic diet or normal Nordic recommendations, with no specific calorie reduction advice.	8 group sessions in first 6 months, plus 4 sessions across 18 months.	At 24 months: −6.2 kg in Paleolithic diet vs. −3.7 kg Normal Nordic recommendations (*p* = NS)	Semi-structured interviews	Thematic analysis
McParlin et al., 2018 [40]	England	Females with gestational diabetes mellitus (*n* = 12)	4 weeks of 1200 kcal/day	Hour-long consultation at baseline and weekly reviews	At 4 weeks: −1.6 ± 1.7 kg. Mean weight change was −0.4 kg/week in the study group vs. +0.3 kg/week in the control group (*p* = 0.002)	Semi-structured interviews	Theoretical Domains Framework
Rehackova et al., 2017 [41]	England	Overweight adults (*n* = 15)	8 weeks of 800 kcal diet using meal replacements	Weekly individual support	At 8−weeks: −14.2 kg (98.0 ± 2.6 to 83.8 ± 2.4 kg, *p* < 0.001)	Semi-structured interviews	Thematic analysis
Studies focusing on weight loss and weight loss maintenance							
Bertz et al., 2015 [42]	Sweden	Postpartum females (*n* = 21)	12-week RCT of calorie-reduced diet (by 500 kcal/day), exercise (45 min brisk walk 4 times per week), diet and exercise, or control.	At baseline and at 6 weeks, plus fortnightly text messages	At 12 weeks: Diet −9.7 ± 4.8% (*p* < 0.001), Diet + Exercise (*p* < 0.001).	Semi-structured interviews	Grounded theory
Brandt et al., 2018 [43]	Denmark	Overweight patients (*n* = 10)	20-month online e-health tool with no specific calorie reduction advice.	4 months of weekly reviews, plus 16 months of optional input	At 4 months: −7.0 kg (*p* < 0.001).	Semi-structured interviews	Thematic analysis
Kleine et al. 2019 [44]	United States of America	Overweight adults (*n* = 61)	8–12 weeks of a proprietary meal replacement programme	20 sessions over 1 year	NA	Focus groups	Content analysis theory
Lawford et al. 2021 [45]	Australia	Adults with osteoarthritis (*n* = 24)	6-month RCT of exercise, exercise plus 800 kcal diet with meal replacements, or control	6 months of monthly virtual consults and access to online resources	NA	Semi-structured interviews	Grounded theory
Metzgar et al., 2015 [46]	United States of America	Overweight and obese females (*n* = 23)	18-week RCT of calorie-reduced diet (by 500 kcal per day) plus energy-controlled chocolate snacks or no chocolate snacks.	18 weeks of weekly group education session	At 18 weeks: −4.4 ± 0.6 kg (*p* < 0.001) in dark chocolate group; −5.0 ± 0.9 kg (*p* < 0.001) in non-chocolate group	Focus groups	Thematic analysis
Östberg et al., 2011 [47]	Sweden	Overweight adults (*n* = 19)	12 weeks of 800 kcal diet using meal replacements, plus 9 months of corset treatment for successful participants.	6 group sessions for 12 week phase, 6 sessions during corset treatment.	85% lost at least 8kg (*p*-value NA)	Focus groups	Grounded theory
Terranova et al., 2017 [48]	Australia	Breast cancer survivors (*n* = 14)	6 months of calorie-reduced diet (by 500 kcal per day) and 210 min of physical activity per week	6 weekly calls, 10 fortnightly calls, and 6 months of tailored text messages	At 6 months: −5.5 kg (*p* < 0.05)	Semi-structured interviews	Thematic analysis
Wycherley et al., 2011 [49]	Australia	Adults with type 2 diabetes (*n* = 30)	16-week RCT of reduced-calorie diet with or without supervised resistance training 3 days per week	Fortnightly individual reviews	At 16 weeks: −8.7% to −12.7% across all interventions (*p* < 0.001)	Semi-structured interviews	Thematic analysis

Abbreviations: RCT = randomised controlled trial, NA = not available.

**Table 2:** Summary of the Critical Appraisal Skills Programme (CASP) judgments.

CASP Question	Number of Answers across All Included Studies		
	Yes	Unclear	No
Was there a clear statement of the aims of the research?	14	0	0
Is a qualitative methodology appropriate?	14	0	0
Was the research design appropriate to address the aims of the research?	13	1	0
Was the recruitment strategy appropriate to the aims of the research?	13	1	0
Was the data collected in a way that addressed the research issue?	14	0	0
Has the relationship between researcher and participants been adequately considered?	12	1	1
Have ethical issues been taken into consideration?	14	0	0
Was the data analysis sufficiently rigorous?	14	0	0
Is there a clear statement of findings?	14	0	0
Is the research valuable?	14	0	0

**Table 3:** Summary of themes derived from the studies reviewed.

Aggregate Dimensions	Second-Order Themes and First-Order Concepts	Supporting Studies
Internal factors	Motivation as a facilitator	
	Drive to improve health	36–42, 45, 47
	High intrinsic motivation	38, 44, 46
	Ready to make a lifestyle change	38, 41, 42, 44
	Weight loss success	36, 38, 45–47
	Experiencing personal benefits	36–39, 41, 47, 48
	Following a structured plan	40, 45–48
	Self-determination	36, 38, 41
	Involvement in a research study	36, 37, 39, 41, 47
	External motivation (e.g., family)	36, 37, 40, 49
	Loss of motivation as a barrier	
	Lack of external accountability/supervision	42, 44, 46, 48
	Slower weight loss	39, 48
	Ambivalence around concrete strategies	38, 41, 45
	Weight loss no longer a priority	36, 45, 46
	Self-efficacy as a facilitator	
	Utilising behaviour regulation strategies	37–39, 41, 44
	Increased perceived self-efficacy	39, 41, 42, 45, 49
	Improved behaviour awareness	40, 42
	Setting clear goals	40, 45, 46–48
	Self-monitoring	42, 45
	Acquiring new knowledge	37, 40, 42
	Low self-efficacy as a barrier	
	Lack of self-regulation	36, 37, 39, 40, 44
	Low self-confidence	36, 37, 39, 44
Programme factors	Acceptability of diet/programme as a facilitator	
	Convenience of diet/programme	39, 41, 47–49
	Programme tools/education facilitated adherence	40, 47, 48
	Flexible restraint	40, 43
	Unacceptability of diet/programme as a barrier	
	Lack of variety/boredom	36, 39, 46–48
	Difficulties transitioning to real food	39, 48
	Too expensive	36, 37, 40, 46
	Did not match taste preferences	37, 39, 46
	Role of study staff as a facilitator	
	Personalised support	36, 38, 39, 42, 47
	Accountability	36, 39, 40, 44, 45, 47, 48
	Trust in/rapport with healthcare professional	38, 40, 42, 43, 46–49
Social factors	Strong social support as a facilitator	
	Group support/shared experience	39–41
	Supportive spouse/family/friends/colleagues	43, 45–47
	Poor social support as a barrier	
	Unsupportive spouse/family/friends/colleagues	41, 44, 46
	Social expectations/pressures	40, 41, 44, 46
	Loss of group support	36, 41, 44
Environmental factors	External factors as a barrier	
	Obesogenic environment	38, 39
	Reduced physical activity opportunities	36, 49
	Reduced capacity to overcome barriers	38, 42, 43, 49

## Data Availability

The datasets used and/or analysed during the current study are available from the corresponding author on reasonable request.
